# H5N1: How prepared are the US and UK for a pandemic?

**DOI:** 10.1371/journal.pgph.0006068

**Published:** 2026-04-08

**Authors:** Samuel Read, Devi Sridhar

**Affiliations:** 1 Edinburgh Medical School, University of Edinburgh, Edinburgh, United Kingdom; 2 Usher Institute, School of Population Health Sciences, University of Edinburgh, Edinburgh, United Kingdom; PLOS: Public Library of Science, UNITED STATES OF AMERICA

Avian influenza (AI), commonly referred to as ‘bird flu’, is an infectious disease which primarily affects populations of wild and domestic birds. Subtypes of this virus are classified as low pathogenicity (LPAI) or high pathogenicity (HPAI), with the latter having a mortality rate of up to 100% in infected chickens [1].

While the majority of H5N1 cases continue to occur in birds, a growing number of mammalian species, including dairy cattle, have started to become infected. Currently, governments, including the UK and US, continue to classify the H5N1 risk to public health as low given its inability to spread between humans. However, recent studies have shown that a single mutation to the virus’ surface protein may be sufficient to switch its receptor specificity from avian to human, meaning that human to human transmission becomes more likely [2].

## Step changes

**Fig 1:**
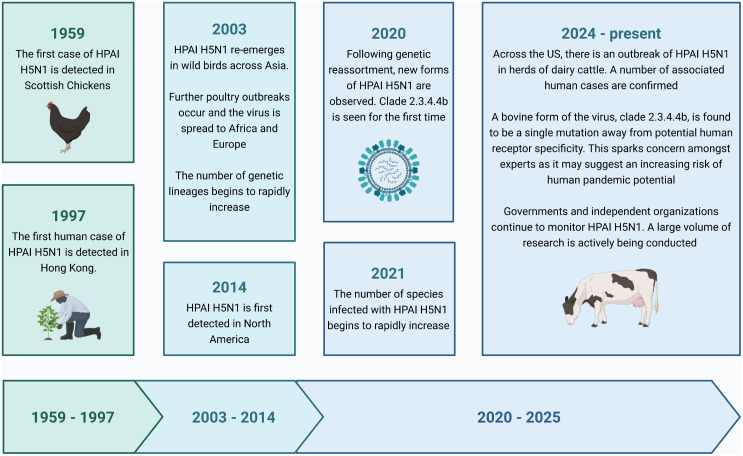
H5N1 step changes. Created in BioRender. Read, S. (2026).

H5N1 was first identified in 1959 during an outbreak in chickens with little attention from public health experts (Fig 1). In 1997, the first human cases of the virus were confirmed, leading to major research investments. These included studies on genetic sequencing, viral pathogenesis in animal model studies, clinical features and rapid viral diagnosis, as well as risk factors for infection [3].

At the end of 2003, the virus re-emerged in wild birds across Asia, predictably leading to further poultry outbreaks and additional human deaths. Wild birds simultaneously facilitated the spread of H5N1 to Africa and Europe, and the number of genetic lineages began to rapidly increase. In 2014, a person returning to Canada was confirmed to be infected with H5N1, marking the first North American case [4].

In 2024, clade 2.3.4.4b of the H5N1 virus became widespread, notably infecting dairy cattle across the US. Soon after, the first likely mammal-to-human transmissions of HPAI H5N1 occurred [5]. Of the cattle-associated human cases of the virus (41 reported officially by the CDC), no one has died and the majority have had mild illness. This is in contrast to other human cases, linked to wild birds, recorded around the same period: the US had its first death from H5N1 in 2024/25 linked to exposure to wild birds (out of a possible 3 cases linked to wild bird exposure), and the one reported human case in Canada (also from wild birds) was severely ill and survived after an extended period in an ICU.

## The United Kingdom

In March 2025, the UK confirmed the first global case of H5N1 in sheep [6]. Following routine testing, a single sheep’s milk tested positive for the virus. While this appears to be an isolated incident, the UK Chief Veterinary Officer has encouraged all animal owners to be aware of the symptoms of avian influenzas [6]. The system to identify cases relies on those in close contact with animals notifying authorities rather than widespread, routine surveillance.

Within the UK, suspected H5N1 case management is initially led locally by the UK Health Security Agency (UKHSA). Expert teams assess and manage any potential risks, escalating to a national incident where necessary [7]. A public health response is initiated when animal culling is recommended, but not generally for human exposures to unconfirmed infections. Containment of confirmed H5N1 outbreaks is undertaken by The Advisory Committee on Dangerous Pathogens, using an approach which emphasises reducing human exposures, prophylactically treating exposed persons with antiviral drugs, and reinforcing PPE guidelines [7].

In 2025, UKHSA published a ‘priority pathogens reference tool’ to allocate funding for research and development for diagnostics, therapeutics, and vaccines [8]. Orthomyxoviridae viruses, the family to which H5N1 belongs, are described as having an overall high pandemic and endemic potential [8]. The UK Government has already secured five million doses of a H5 vaccine and an advanced purchase agreement for further doses as needed: this is for a generic vaccine and not tailored to the specific strain of H5N1 that is spreading or could spread in humans [9].

## The United States

Management of H5N1 in the US is overseen by the US Centres for Disease Control and Prevention, the US Department of Agriculture, and the US Department of Health and Human Services. Planning and response to H5N1 in the US had been broadly similar to in the UK until 2025. For at risk groups, such as those who work with animals, control measures have been put in place and information regarding risks has been clearly shared. Those who become exposed to H5N1 are isolated and monitored for a period of 10-days, simultaneously taking a course of antiviral drugs such as TamiFlu [10].

Prior to 2025, the US government had announced a $590 million investment to advance the development of a H5N1 vaccine. However, recent changes from the Trump Administration mean the specifics of this investment have since been reevaluated [11]. The current US Secretary of Health and Human Services, Robert F. Kennedy Jr., has suggested that the H5N1 virus should be allowed to spread through flocks of chickens to allow farmers to identify birds which may be immune. This position, against the advice of experts, marks a significant change in government strategy [12].

Key health agencies in the US have lost a significant number of employees. As part of the ‘make America healthy again’ agenda which emphasizes chronic conditions over infectious disease, the Department of Health and Human Services has announced a ‘dramatic restructuring’ with a resultant loss of approximately 20,000 full-time staff members [13]. Additionally, the Department for Agriculture has offered financial incentives for key personnel to quit [14] and been criticised for not providing detailed and up-to-date data surrounding H5N1 in dairy cattle. This means we’re largely flying blind, not knowing whether or where testing is taking place, what herds are infected and what broader US government policy is to respond to isolated human cases or larger outbreaks.

## Conclusion

What you don’t look for, you don’t find…until it’s too late. We’re not hearing about H5N1 right now, not because it’s gone away, but because of the current US government’s approach to public health. Ongoing silent spread in dairy cattle makes the country a likely location for an outbreak of human-to-human transmission. The timing couldn’t be worse given the Trump administration’s funding cuts for scientific research, vaccine-hesitant position, and general lack of transparency and gaps in information-sharing.

Systems surrounding H5N1 preparations in the UK are generally more stable. While cuts to foreign aid are scheduled to come into effect 2027, these are relatively minor compared to what has been seen in the US. Moreover, funding for specific monitoring and research regarding H5N1 appears to be better protected. The UK Government must, however, remain vigilant to the ever-evolving threat posed by the virus, and continue to support both national and international efforts.

We can only hope that H5N1 does not mutate to allow human-to-human transmission in the current political atmosphere, and that we have sufficient time and resources to prepare for a pandemic. If the virus does make this jump, it could be disastrous for global human health, especially if it maintains its high mortality rate in humans infected by wild birds. Projections range from (more conservative) 2–7 million deaths to more extreme scenarios of hundreds of millions depending on infection spread and available medical counter-measures. We know from recent experience a pandemic isn’t a hypothetical. We must fully plan and consider for all possible outcomes going forward.
